# Genomic insights into the spread of methicillin-resistant *Staphylococcus aureus* involved in ear infections

**DOI:** 10.1186/s12879-025-11052-9

**Published:** 2025-05-06

**Authors:** Zhewei Sun, Jinhong Chen, Chunhong Liu, Yueru Tian, Fuqi Ai, Jiaying Du, Wangxiao Zhou, Wenjun Cao, Ming Guan, Baixing Ding

**Affiliations:** 1 Department of Laboratory Medicine, Shanghai Medical College, Huashan Hospital, Fudan University, Shanghai, 200040 China; 2 Shanghai Institute of Infectious Disease and Biosecurity, Fudan University, Shanghai, 200040 China; 3 Department of Clinical Laboratory, Eye & ENT Hospital, Shanghai Medical College, Fudan University, Shanghai, 200040 China; 4 Clinical Laboratory Center, The Second Affiliated Hospital & Yuying Children’s Hospital of Wenzhou Medical University, Wenzhou, Zhejiang China; 5 Institute of Antibiotics, Shanghai Medical College, Huashan Hospital, Fudan University, Shanghai, 20040 China

**Keywords:** Methicillin-resistant staphylococcus aureus, Ear infections, Transmission, Antimicrobial resistance, Whole genome sequencing

## Abstract

**Background:**

Methicillin-resistant *Staphylococcus aureus* (MRSA) is a major pathogen causing ear infections. However, genomic epidemiology and determinants influencing transmission of ear infections associated MRSA (EIA-MRSA) in community remain unknown.

**Methods:**

In 2020–2021, 105 EIA-MRSA isolates were collected and sequenced from outpatients across different households in Shanghai, China. Antimicrobial susceptibility testing, core genome MLST, and phylodynamic analyses were conducted to characterize EIA-MRSA dissemination.

**Results:**

Quinolone resistance was identified as a risk factor for EIA-MRSA spread (OR 9, [95% CI 3–31]). The ST764 clone and two subclones of ST22-PT hypervirulent clone have developed an extensive quinolone-resistant (eQR) phenotype, conferring additional resistance to advanced quinolones due to the accumulation of four mutations in *gyrA* (S84L and either S85P, E88K, or E88G) and *parC* (S80F and either E84K or E84G). These ST764- and ST22-PT-eQR isolates were highly transmissible and showed increased resistance to other commonly used antimicrobials, posing potential high-risk clones. The eQR phenotype may be inherent to the ST764 lineage, which emerged in the late 1980s, coinciding with the widespread fluoroquinolone usage. The ST22-PT-eQR subclones emerged in around 2017 and are accumulating resistance genes.

**Conclusion:**

Vigilance is crucial for eQR high-risk clones, particularly the convergent ST22-PT-eQR subclones that accumulate resistance and virulence traits, posing risks for ear infections.

**Clinical trial number:**

Not applicable.

**Supplementary Information:**

The online version contains supplementary material available at 10.1186/s12879-025-11052-9.

## Introduction

Ear infections are a common global health issue that can lead to hearing loss and even neurological complications [1]. Such infections are estimated to cost billions of dollars annually in the United States [2]. *Staphylococcus aureus* is one of the major bacterial pathogens responsible for ear infections worldwide [3]. Importantly, Methicillin-resistant *S. aureus* (MRSA) is often multidrug-resistant and more prone to developing resistance to commonly used antimicrobials [4], which complicates infections compared to other bacterial pathogens. Therefore, understanding the genomic epidemiology and factors that contribute to the spread of ear infections associated MRSA (EIA-MRSA) in the community is essential for developing effective intervention strategies.

Recent advancements in whole-genome sequencing (WGS) have deepened our understanding of MRSA populations and transmission dynamics [5]. However, genomic studies specifically focusing on MRSA involved in ear infections are currently lacking.

In this study, we reported a cohort of 105 EIA-MRSA isolates collected from outpatients across different households. Importantly, we found that quinolone resistance is significantly correlated with the clonal dissemination of EIA-MRSA. High-risk clones, such as the ST764 clone and two subclones of the hypervirulent ST22-PT (which carry both Panton-Valentine leucocidin [PVL] and toxic shock syndrome toxin 1 [TSST-1]), have acquired an extensively quinolone-resistant (eQR) phenotype, exhibiting resistance to both classic and advanced quinolones. These eQR clones demonstrate enhanced transmissibility within the community and increased resistance to other commonly used antibiotics. The emergence of eQR high-risk clones poses a greater burden on ear infections, necessitating strengthened surveillance and control measures for these strains.

## Materials and methods

### Inclusion criteria for EIA-MRSA isolates and whole-genome sequencing

The study initially included 109 MRSA isolates cultured from ear discharge specimens of non-duplicated outpatients with aural inflammation between January 2020 and December 2021 at the Eye & ENT Hospital, Fudan University, Shanghai, China. Presence of *mecA* was confirmed by polymerase chain reaction. To investigate EIA-MRSA transmission in the Shanghai community, all patients were confirmed to be local residents with no household history of ear infections, thereby excluding the possibility of intra-household transmission. Finally, 105 EIA-MRSA isolates were eligible for downstream analysis. Genomic DNA from 105 EIA-MRSA isolates was extracted using Gentra Puregene Yeast/Bact. Kit (Qiagen, San Francisco/Bay Area, CA, USA). WGS was performed on an Illumina novaseq 6000 platform (Illumina, San Diego, CA, USA) with 2 × 150-bp paired-end libraries.

### Transmission analysis of EIA-MRSA isolates

We used a *S. aureus* core genome multilocus sequence typing (cgMLST) scheme [6] to determine the genetic relatedness between isolates. Two isolates differing by fewer than 12 alleles in the core genome were considered genetically related [6]. The network function in the R package network v1.17.1 was used to construct the transmission network and extract clusters of connected nodes (isolates).

### Antimicrobial susceptibility testing

The minimum inhibitory concentrations (MICs) of EIA-MRSA isolates were determined using the broth microdilution method. Seventeen antimicrobials were tested, including levofloxacin, nemonoxacin, sitafloxacin, penicillin, cefoxitin, oxacillin, vancomycin, teicoplanin, gentamicin, amikacin, azithromycin, erythromycin, clindamycin, trimethoprim-sulfamethoxazole, rifampin, linezolid, and nitrofurantoin. Susceptibility and resistance phenotypes were defined based on MIC cutoffs according to the Clinical and Laboratory Standards Institute [7], except for the advanced quinolones nemonoxacin and sitafloxacin. Nemonoxacin resistance was defined as MIC ≥ 2 µg/mL [8], and sitafloxacin resistance followed ciprofloxacin cutoff values, with MIC ≥ 4 µg/mL considered resistant [9]. Raw MIC values were provided in Supplementary Dataset S1 (Excel Sheet 1).

### Phylogenetic analysis

Core gene alignment of 105 EIA-MRSA isolates were construct using Panaroo v.1.5.0 [10]. Maximum likelihood (ML) tree were generated with RAxML v8.1.20 [11] using core gene alignment as input. Eighty-three ST764 MRSA and 1,042 ST22 *S. aureus* public genomes were downloaded from NCBI RefSeq (see Supplementary Dataset S1, Excel Sheet 2). Non-recombined core genome alignments of ST764 and ST22 were generated using Snippy v4.6.0 (https://github.com/tseemann/snippy) and Gubbins v3.3.5 (https://github.com/nickjcroucher/gubbins). A ML tree of the ST22 lineage was generated with RAxML using core genome alignment as input, with strain SA28-SX (CP130515) as the reference. Time-calibrated phylogenies of the ST764 and ST22-PT clones were constructed using BEAST v1.10.4 [12] with a strict molecular clock. Bayesian skyline and constant-size models were used for the ST764 and ST22-PT clones, respectively. Three independent runs of 50 million MCMC generations were performed, with samples taken every 5000 generations. Log files were inspected in Tracer v1.7.1 (https://github.com/beast-dev/tracer) for convergence, mixing, and sufficient sampling.

### Statistical analysis

All statistical analyses were conducted using Python (v3.8.6). Specific tests used in this study are given together with each result in the text. The corresponding functions in the scipy.stats package are as follows: chi2_contingency for the Chi-square test (two-sided); fisher_exact for Fisher’s exact test (two-sided); mannwhitneyu for the Wilcoxon rank-sum test (two-sided). Odds ratios (ORs) and 95% confidence intervals (CIs) were computed using the Table 2 × 2 class from the statsmodels Python package, based on 2 × 2 contingency tables.

## Results

To investigate the clonal diversity of EIA-MRSA, a ML tree was constructed from 105 isolates in this study. Eleven clonal complexes (CCs) were identified, with the majority of isolates found in CC59 (35/105), CC5 (21/105), CC398 (16/105), CC1 (9/105), and CC22 (8/105) (Figure S1).

### Quinolone resistance promotes community spread of EIA-MRSA

**Fig. 1:**
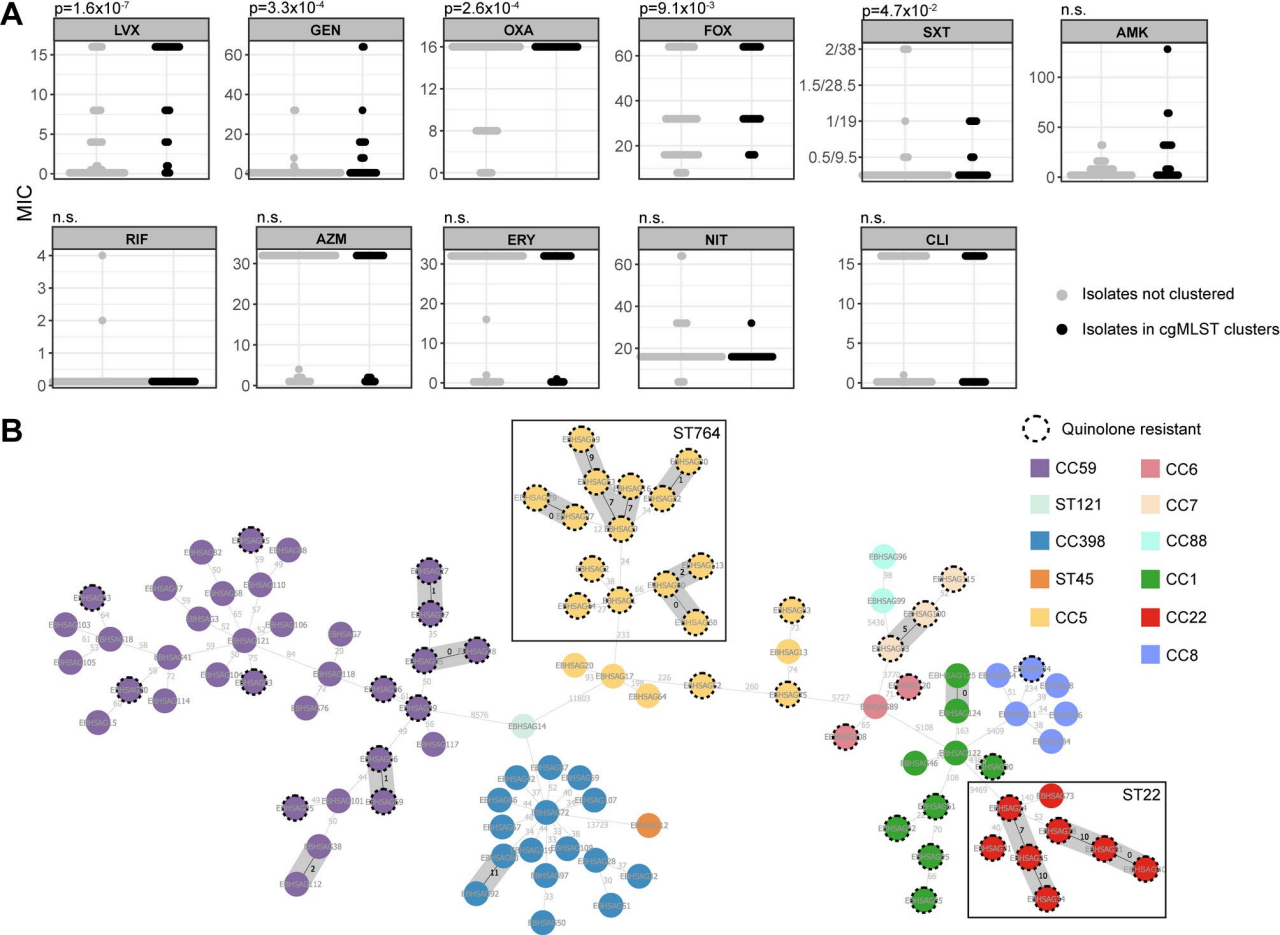
Comparison of MICs for different antimicrobials between genetically related (i.e., isolates in cgMLST clusters) and unrelated EIA-MRSA isolates. (**A**) Distribution of MICs in genetically related (black dots) and unrelated (grey dots) EIA-MRSA isolates across different antimicrobials. (**B**) Minimum spanning tree based on the core genome of 105 EIA-MRSA isolates. Node colors represent the clonal complex (CC). Thirteen cgMLST clusters, each with at least two isolates and a maximum of eleven allelic differences, are highlighted with a grey background. Dashed outlines around nodes indicate isolates resistant to levofloxacin. Allelic differences between two isolates are indicated on the edges. ST764 and ST22 isolates are enclosed in boxes

Acquisition of increased AMR is a key adaptive advantage for the spread of pathogens. We identified 13 cgMLST clusters comprising genetically-related isolates, totaling 31 isolates (2–4 per cluster). We subsequently compared the minimum inhibitory concentrations (MICs) of 11 commonly used antimicrobials between the genetically-related isolates (i.e., those within cgMLST clusters, *n* = 31) and the genetically-unrelated isolates (*n* = 74). The results revealed that genetically-related isolates exhibited significantly higher MICs for levofloxacin (*p* = 1.6 × 10^− 7^), gentamicin (*p* = 3.3 × 10^− 4^), oxacillin (*p* = 2.6 × 10^− 4^), cefoxitin (*p* = 9.1 × 10^− 3^), and trimethoprim-sulfamethoxazole (*p* = 4.7 × 10^− 2^) (Fig. 1A). After mapping the quinolone-resistant and susceptible phenotypes onto the cgMLST tree, we confirmed that quinolone-resistant isolates are more likely to spread clonally in the community (OR 9, [95%CI 3–31], *p* = 3.8 × 10^− 6^) (Fig. 1B).

### ST764 and ST22 EIA-MRSA isolates exhibit extensive quinolone resistance

**Fig. 2:**
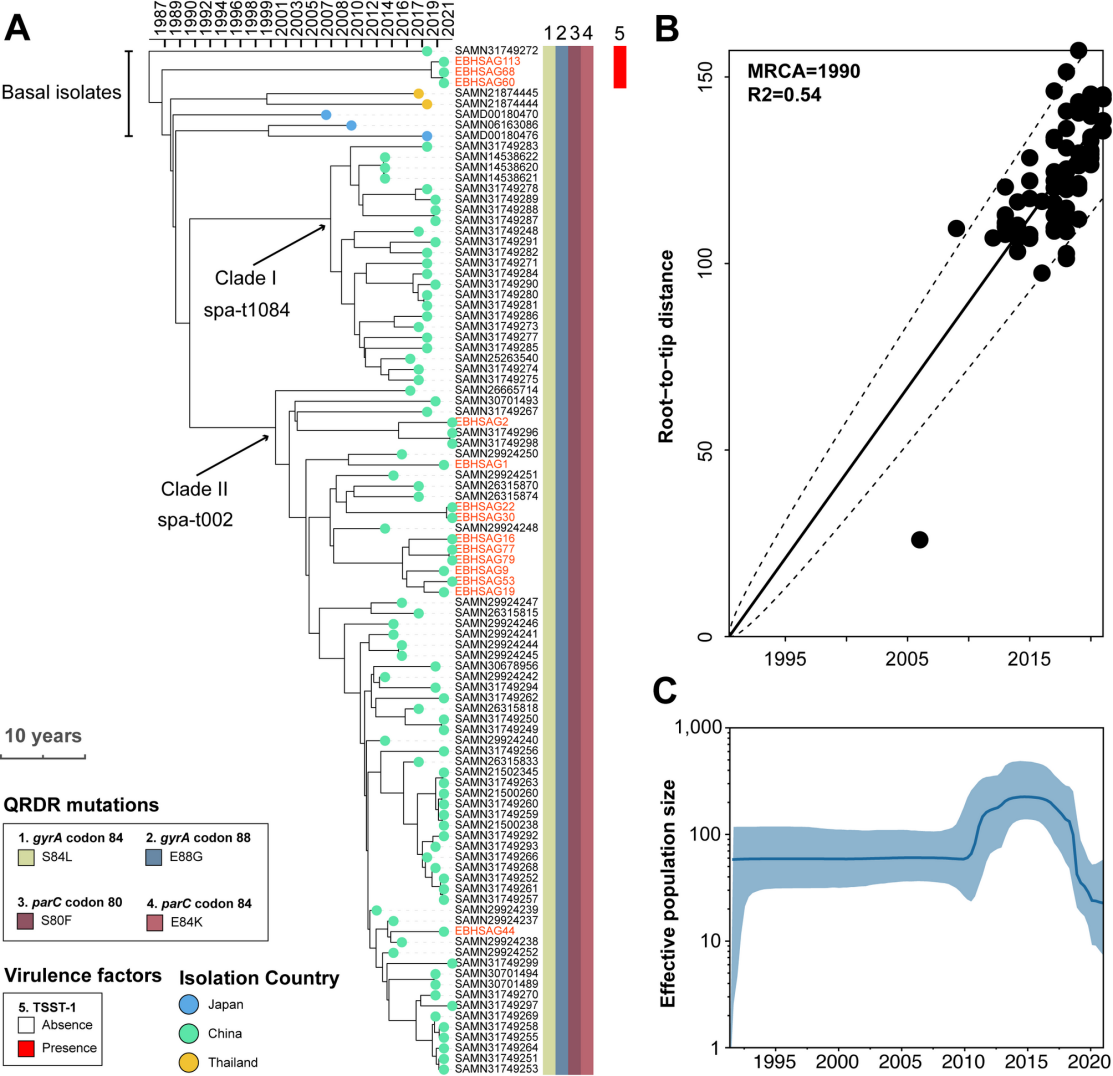
Temporal phylogenetic reconstruction of ST764 MRSA lineage. (**A**) Bayesian phylogenetic reconstruction of the ST764 MRSA lineage using 1,684 non-recombinant core genome SNPs from 97 isolates (14 from this study). Isolates are represented by dots at the tree tips, colored by isolation country. Strain names of EIA-MRSA isolates are colored in red. Heatmaps show the presence of QRDR mutations and the TSST-1 virulence factor, with colored cells indicating presence and white cells indicating absence. (**B**) Linear regression plot showing the relationship between date (x-axis) and root-to-tip divergence (y-axis), used to assess the correspondence between the phylogenetic signal (core genome SNPs-based maximum likelihood phylogeny of the ST764 MRSA lineage) and temporal signal (isolation date). (**C**) Bayesian skyline plot showing changes in the effective population size of the ST764 MRSA population (*n* = 97) over time. The estimated variations are represented by a blue line, with 95% confidence intervals depicted by a blue shaded area

To assess the efficacy of more advanced quinolones against EIA-MRSA, we additionally tested the resistance of 105 EIA-MRSA isolates to sitafloxacin and nemonoxacin. Nemonoxacin and sitafloxacin are the newer generation quinolones, respectively, approved in China in 2016 [13] and 2019 [14]. The results indicate that none of the levofloxacin-sensitive EIA-MRSA isolates were resistant to sitafloxacin or nemonoxacin (Figure S2). Among the 48 levofloxacin-resistant isolates, 19 remained resistant to sitafloxacin (Figure S2) and 21 to nemonoxacin (Figure S2). Notably, all isolates exhibiting the extensively quinolone-resistant (eQR) phenotype—defined as resistance to levofloxacin, sitafloxacin, and nemonoxacin—belonged to either the CC5-ST764 (14/14) or CC22-ST22 (5/8) lineages (Fig. 2C). All eQR isolates acquired four QRDR mutations in *gyrA* (S84L and either S85P, E88K, or E88G) and *parC* (S80F and either E84K or E84G) (Figure S1). For example, two ST22 EIA-MRSA strains, EBHSAG24 and EBHSAG25, were genetically related (differing by 7 cgMLST alleles). EBHSAG25 acquired additional *gyrA* S85P and *parC* E84 mutations compared to EBHSAG24, resulting in additional resistance to sitafloxacin and nemonoxacin. Interestingly, mutations at codon 84 in *parC* appear to be essential for the eQR phenotype, with 100% sensitivity and specificity. Specifically, all 19 eQR isolates acquired *parC* E84K or E84G mutations, whereas none of the 86 non-eQR isolates had mutations at this codon (Figure S1).

Notably, ST764 and ST22 eQR isolates were highly transmissible compared to the dominant Chinese clone CC59 (OR 12, [95% CI 2–81] and OR Inf, [95% CI 2–Inf], respectively) and showed increased resistance to other commonly used antimicrobials (Table S1).

### The eQR phenotype is inherent to the ST764 lineage

In our dataset, all 14 ST764 EIA-MRSA isolates exhibited the eQR phenotype (Figure S2), with 11 of the 14 isolates grouped into three cgMLST clusters (Fig. 1B). This suggests that the ST764 lineage may have an increased propensity to spread within the community and cause ear infections. A time-calibrated phylogeny of 97 ST764 MRSA isolates (83 isolates from NCBI RefSeq) revealed that the ST764 lineage emerged in 1985 (95% CI, 1979–1990) (Fig. 2A). A correlation (R² = 0.54) between root-to-tip branch lengths and isolation dates supports the robustness of the dating analysis (Fig. 2B). All ST764 isolates carried SCCmec type IIa. Approximately 95% of the ST764 isolates were isolated in China. The phylogeny consisted of two major clades [Clade I, spa-t1084 (*n* = 23, emerged around 2007) and Clade II, spa-t002 (*n* = 65, emerged around 2000)], and the rest 9 isolates found in basal position. EIA-MRSA isolates were primarily clustered in Clade II (11/14), while the remaining three were positioned basally (Fig. 2A). Notably, all ST764 isolates had accumulated four QRDR mutations, including *parC* E84K, suggesting that all ST764 isolates may exhibit the eQR phenotype. All ST764 isolates were negative for Panton-Valentine leukocidin (PVL). However, four basal isolates, including three EIA-MRSA isolates, harbored toxic shock syndrome toxin 1 (TSST-1). Bayesian skyline analysis indicated ST764 MRSA experienced a slight population expansion (threefold) during 2010–2013, followed by a sharp decrease (tenfold) after 2016. (Fig. 2C)

### ST22 EIA-MRSA isolates with eQR phenotype emerge from ST22-PT hypervirulent clone

**Fig. 3:**
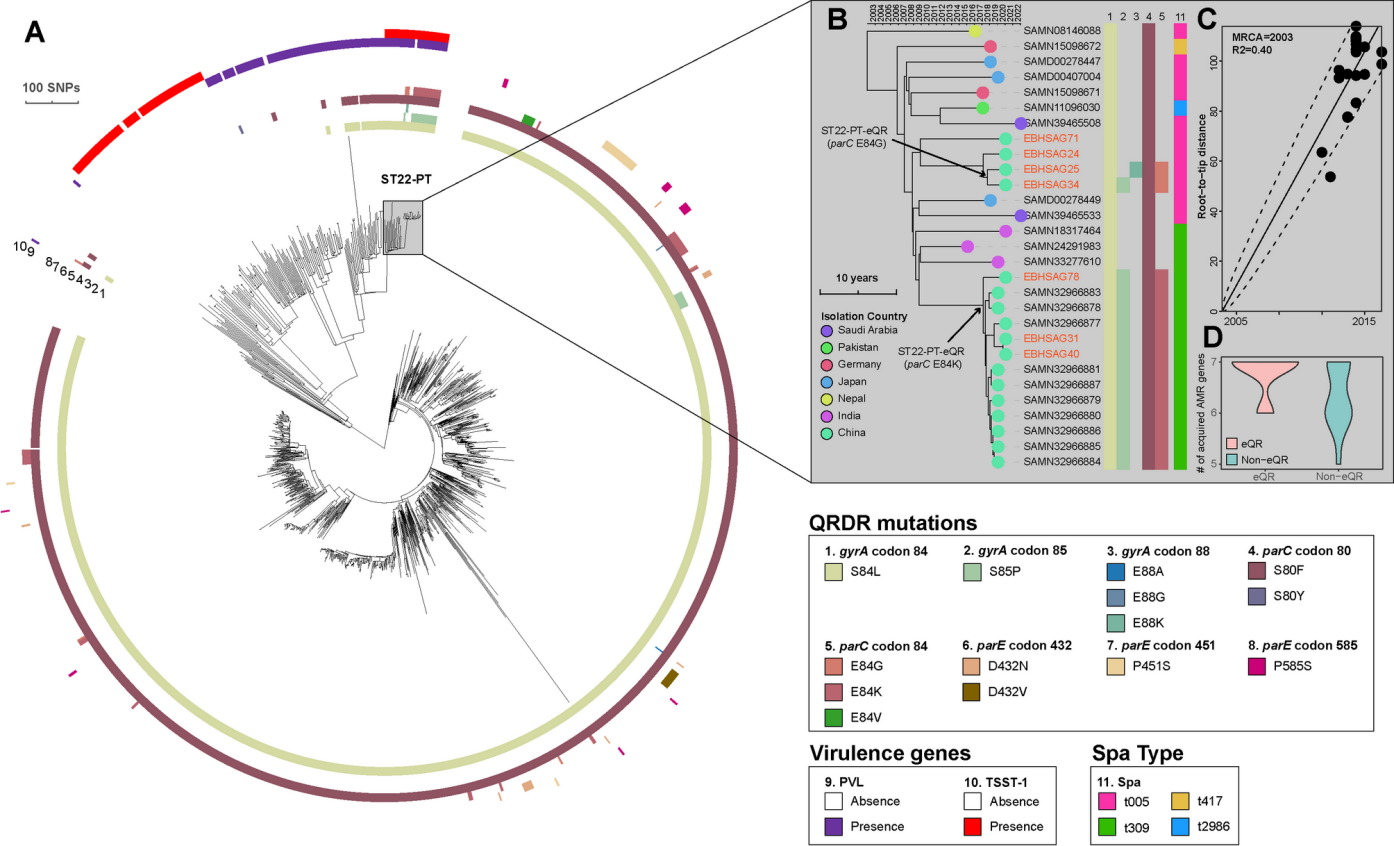
Maximum-likelihood phylogeny of the ST22 *S. aureus* lineage and temporal phylogenetic reconstruction of the ST22-PT hypervirulent MRSA clone. (**A**) Maximum-likelihood tree of 1,051 ST22 isolates (8 from this study) based on 21,413 non-recombinant core genome SNPs. Rings 1 to 8 (from inside to outside) indicate the presence of QRDR mutations, with colored cells indicating presence and white cells indicating absence. Rings 9 and 10 indicate the presence of PVL and TSST-1 virulence factors, respectively. The tree is midpoint-rooted, and the scale bar represents the number of substitutions. (**B**) Bayesian phylogenetic reconstruction of the ST22-PT hypervirulent MRSA clone using 818 non-recombinant core SNPs from 29 ST22-PT isolates (7 from this study). Isolates are represented by dots at the tree tips, colored by isolation country. Strain names of EIA-MRSA isolates are colored in red. (**C**) Linear regression plot showing the relationship between date (x-axis) and root-to-tip divergence (y-axis), used to assess the correspondence between phylogenetic and temporal signals. (**D**) Comparison of the number of acquired antimicrobial resistance genes in ST22-PT-eQR isolates versus ST22-PT isolates without eQR genotypes

Aside from ST764 EIA-MRSA, we also found that ST22 EIA-MRSA is also highly transmissible, with 6 out of 8 isolates falling into two cgMLST clusters (Fig. 1B), and 5 out of 8 exhibiting the eQR phenotype (Figure S2). After placing 8 ST22 EIA-MRSA isolates into a global context by including 1,042 ST22 isolates from NCBI RefSeq, we found that 7 out of 8 EIA-MRSA isolates belonged to the recently recognized ST22-PT hypervirulent MRSA clone, which carries both PVL and TSST-1 [15] (Fig. 3A). All 29 ST22-PT isolates (22 of which were from NCBI RefSeq) carried SCCmec type IVa. Two major spa types were identified in the ST22-PT clone: t005 (16/29, 55.2%) and t309 (11/29, 37.9%) (Fig. 3B).Among the ST22-PT isolates, 15 had accumulated four QRDR mutations, including E84K or E84G in *parC*, suggesting that these isolates may have acquired the eQR phenotype (hereafter ST22-PT-eQR) (Fig. 3B). The ST22-PT clone is estimated to have emerged in 2002 (95% CI, 1993–2008). A sufficient temporal signal was observed in the ST22-PT clone (R² = 0.4) (Fig. 3C). ST22-PT-eQR isolates with the *parC* E84G mutation include only two EIA-MRSA isolates, making the transmission of this subclone not traceable. However, ST22-PT-eQR isolates with *parC* E84K substitution may have disseminated clonally in China since 2017 (95% CI, 2016–2018) (Fig. 3B), with isolates in this subclone exhibiting a pairwise core SNP distance ranging from 3 to 22 (median, 13.5). This eQR subclone consists of isolates from Hubei and Shanghai provinces (three EIA-MRSA isolates), which are approximately 900 km apart, providing evidence of interprovincial transmission. Interestingly, ST22-PT-eQR isolates harbored a greater load of AMR genes compared to other ST22-PT isolates (median 7 vs. 6, *p* = 0.04) (Fig. 3D and S3), indicating that, in addition to accumulating QRDR mutations, resistance genes are also accumulating in ST22-PT-eQR isolates.

## Discussion

*S. aureus* is a leading pathogen responsible for ear infections. Recently, the emergence of MRSA, particularly convergent clones that concurrently accumulate resistance and virulence factors, has posed significant challenges to the treatment of community-acquired infections. Here, a genomic analysis of 105 community-acquired EIA-MRSA isolates was performed to identify the characteristics of MRSA linked to ear infections. We demonstrated that quinolone resistance is a key driver of the spread of EIA-MRSA in the community. The highly transmissible ST764 clone and two subclones of the ST22-PT hypervirulent clone were identified as having developed the eQR phenotype, representing potential high-risk clones for causing ear infections.

An important goal of this study is to identify the factors facilitating the spread of MRSA in community-acquired ear infections. Identifying the characteristics of highly transmissible clones responsible for ear infections in the community can provide a basis for rapid targeted therapy against these clones. Interestingly, combining genomic analysis with antimicrobial susceptibility testing results, we found that quinolone-resistant isolates were more likely to undergo clonal dissemination. As quinolones are the first-line treatment for ear infections, the acquisition of quinolone resistance undoubtedly provides a survival and transmission advantage for EIA-MRSA in this context. A similar pattern was observed in our previous study on *Pseudomonas aeruginosa* associated with ear infections [16]. Strikingly, testing the resistance of EIA-MRSA isolates to two advanced quinolones, nemonoxacin and sitafloxacin, revealed that the ST764 clone and two subclones of ST22 EIA-MRSA had developed the eQR phenotype. Moreover, the eQR EIA-MRSA isolates exhibited significantly higher MICs to β-lactams, aminoglycosides, and lincosamides compared to non-eQR EIA-MRSA (Table S1). Transmission analysis also indicated that eQR EIA-MRSA isolates of ST764 and ST22 have a greater likelihood of clonal dissemination within the community. This suggests that antibiotic treatment options for ear infections caused by these eQR MRSA isolates will be more limited.

ST764 MRSA is classified as a hybrid variant of ST5 MRSA that emerged recently [17]. A previous study revealed that two major subclones of ST764 MRSA, ST764-t1084 (Clade I) and ST764-t002 (Clade II) (Fig. 2A), were circulating in China. ST764-t1084 isolates are hypervirulent but exhibit lower adhesion abilities compared to ST764-t002 hypovirulent isolates [17]. Despite comprehensive profiling of virulence-associated genes, no significant differences were detected between ST764-t002 and ST764-t1084 clones. This suggests that the observed phenotypic divergence in adhesion and biofilm formation may originate from post-transcriptional regulation (e.g., quorum sensing) [18], novel virulence determinants or unannotated genetic elements (e.g., small RNAs) [19], rather than canonical virulence gene repertoires. We found that 11 out of the 14 ST764 EIA-MRSA isolates belonged to the ST764-t002 subclone (Clade II), while none of the EIA-MRSA isolates belonged to the ST764-t1084 subclone. This suggests that ST764-t002 isolates may be better adapted to the ear environment, potentially due to their higher adhesion abilities. However, it is concerning that all ST764 isolates, including those from outside China, have accumulated four QRDR mutations, including the *parC* E84K mutation. This makes ST764 MRSA a potential high-risk clone for causing ear infections. Our Bayesian analysis suggested that the ST764 MRSA clone emerged in the late 1980s and experienced a slight population expansion (threefold) during 2010–2013, followed by a tenfold decrease after 2016 (Fig. 2C). The emergence of this eQR MRSA clone coincided with the widespread use of fluoroquinolones starting in the late 1980s [20]. The decline in the effective population size of ST764 from 2016 onwards may be a consequence of the recent reinforcement of antibiotic management policies by the Chinese government in 2016 [21].

Perhaps even more concerning than the emergence of MDR pathogens is the rise of convergent pathogens—those that acquire both hypervirulence and MDR phenotypes. Notably, our study reveals that the recently recognized ST22-PT hypervirulent MRSA clone is accumulating antimicrobial resistance. All ST22 EIA-MRSA isolates with the eQR phenotype in this study were identified as belonging to the ST22-PT clone (Fig. 3B). Furthermore, our analyses suggest that the ST22-PT-eQR subclones emerged around 2017, with the subclone harboring *parC* E84K showing signs of interprovincial dissemination within mainland China. These ST22-PT-eQR isolates also possessed a higher number of acquired AMR genes (Fig. 3D). Therefore, the community spread of ST22-PT-eQR subclones represents a significant public health threat, not only for ear infections but also for other community-acquired infections. Heightened vigilance and control measures are needed to combat the risks posed by these convergent MRSA isolates. Although MRSA is a well-known pathogen with a rising incidence in ear infections, genomic epidemiological studies on MRSA causing ear infections are currently lacking. Our study benefits from a targeted focus on MRSA isolates sampled from outpatients with ear infections. However, we limited our analysis to MRSA, despite the growing importance of methicillin-susceptible *S. aureus* (MSSA) in causing ear infections. Since the epidemiology of MSSA and MRSA differs profoundly in terms of colonization, infection, and transmission [22], our results may not be generalizable to MSSA.

In conclusion, our work characterizes the transmission dynamics of community-acquired MRSA associated with ear infections. The adaptive evolution of EIA-MRSA indicates that preferential antibiotic use has led to the emergence of high-risk clones demonstrating remarkable adaptation to antimicrobial selection pressures. This worrisome situation calls for urgent enhancement and sustained maintenance of global antimicrobial stewardship standards to prevent the repeated emergence of additional MDR or even more formidable convergent clones.

## Electronic supplementary material

Below is the link to the electronic supplementary material.

Supplementary Material 1

Supplementary Material 2

## Data Availability

Short reads data of the 105 EIA-MRSA isolates in this study have been deposited in the NCBI SRA database under BioProject PRJNA1176299.
