# Supplementary material for: Genomic insights into the spread of methicillin-resistant Staphylococcus aureus involved in ear infections
Source: BMC Infect Dis. 2025 May 6;25:661. doi: 10.1186/s12879-025-11052-9 (PMC12054198; doi:10.1186/s12879-025-11052-9)
Supplement: Supplementary file 2 — Supplementary Material 2 [file 12879_2025_11052_MOESM2_ESM.pdf]

Supplementary Figures

Figure S1: Phylogenetic inference of the 105 EIA-MRSA isolates. Maximum-likelihood tree based on 1,939 concatenated core genes conserved in 100% of EIA-MRSA genomes. Dots at the tree tips indicate quinolone resistance: blue represents isolates resistant to levofloxacin only; teal represents isolates resistant to both levofloxacin and nemonoxacin, but not sitafloxacin; orange represents isolates with the eQR phenotype, resistant to levofloxacin, nemonoxacin, and sitafloxacin. Tree tips without dots represent quinolone-sensitive isolates. The heatmap shows the clonal complex (CC) of the isolates and the presence of QRDR mutations (colored cells indicating presence and white cells indicating absence).

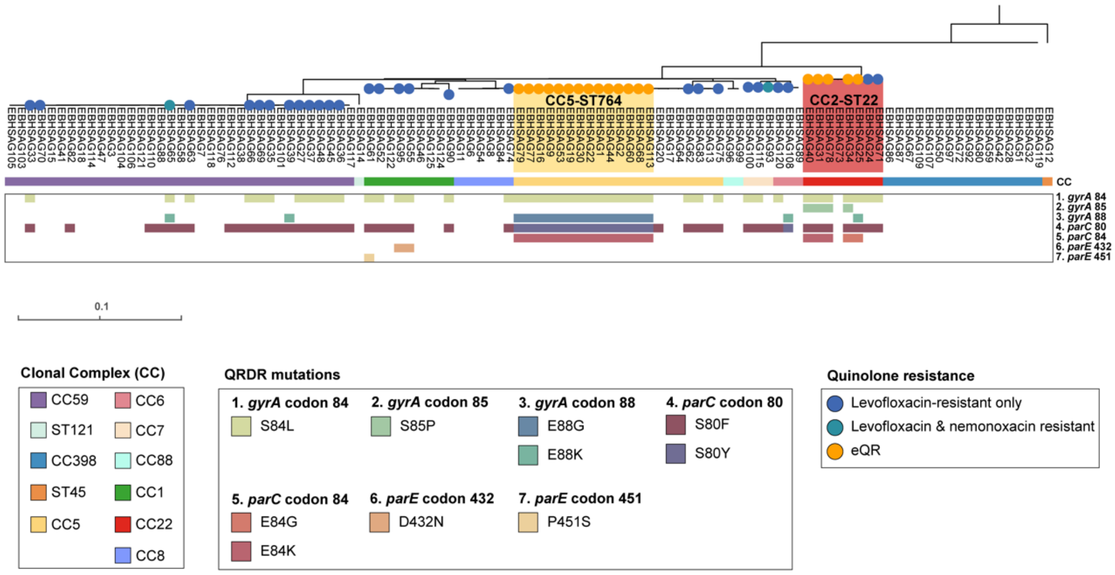

Figure S2: Alluvial plot showing the relationship between levofloxacin resistance and resistance to sitafloxacin or nemonoxacin in EIA-MRSA isolates. (A) Relationship between levofloxacin and sitafloxacin resistance in EIA-MRSA isolates. (B) Relationship between levofloxacin and nemonoxacin resistance in EIA-MRSA isolates. (C) Relationship between clonal complexes of the 48 levofloxacin-resistant EIA-MRSA isolates and their resistance to nemonoxacin and sitafloxacin.

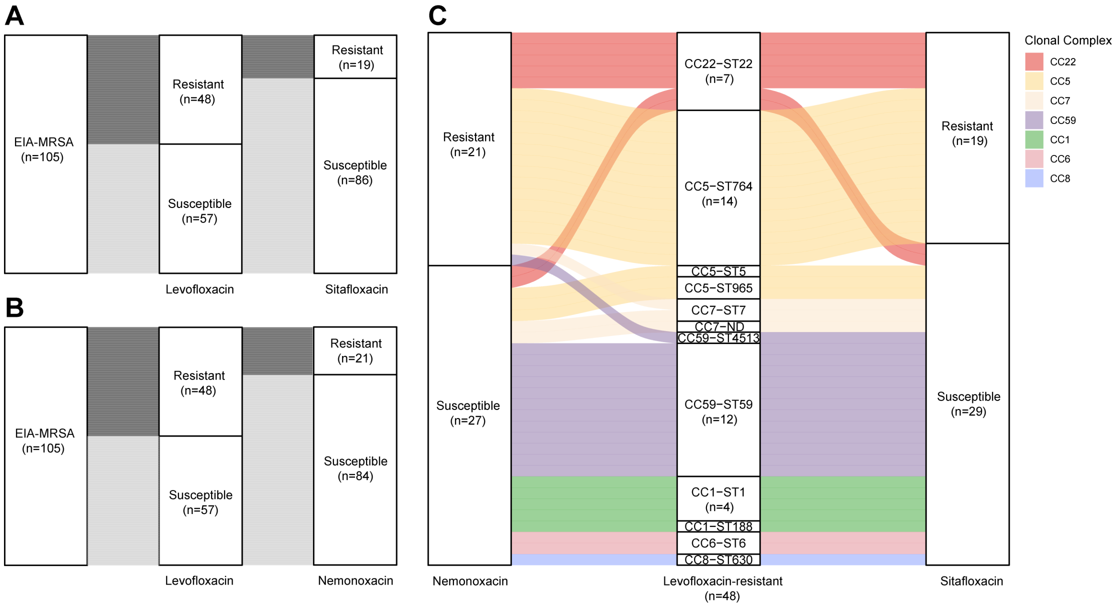

Figure S3: Distribution of antimicrobial resistance genes and virulence genes in the ST764 and ST22-PT clones. (A) Distribution of antimicrobial resistance genes in the ST764 clone; (B) Distribution of virulence genes in the ST764 clone; (C) Distribution of antimicrobial resistance genes in the ST22-PT clone; (D) Distribution of virulence genes in the ST22-PT clone. EIA-MRSA strains are highlighted in red font.

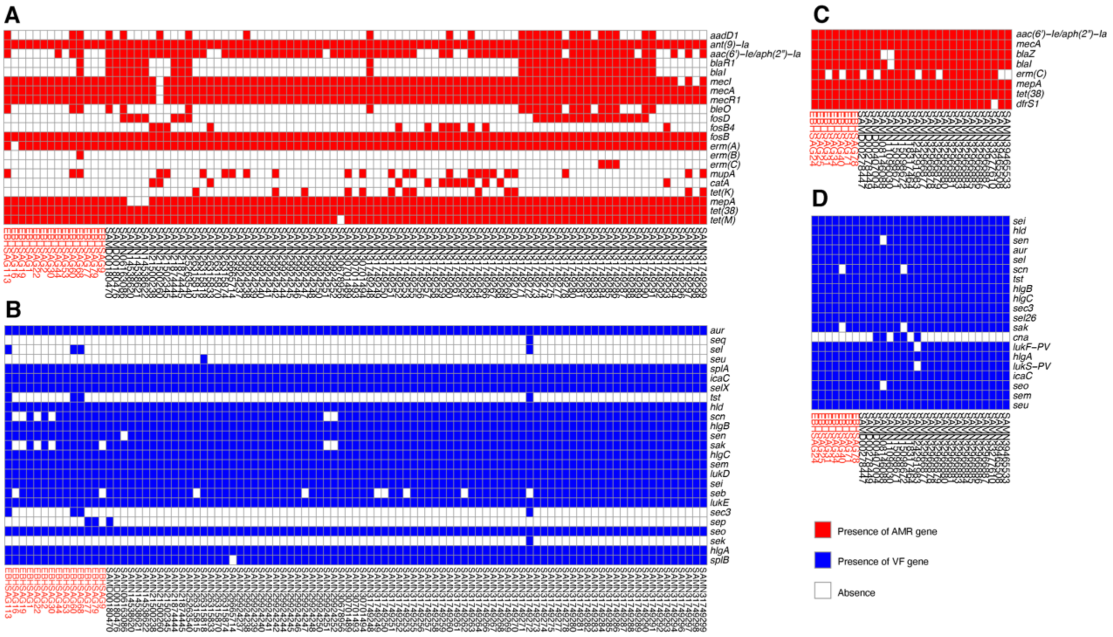

Supplementary Tables

Table S1: Comparison of MIC_{50} values for 10 non-quinolone drugs between EIA-MRSA isolates with and without the eQR phenotype.

Drug	MIC_{50} (eQR)	MIC_{50} (Non-eQR)	P value
FOX	64	32	0.001
OXA	16	16	0.06
GEN	0.5	0.5	0.05
AMK	8	2	0.04
AZM	32	32	0.09
ERY	32	32	0.08
CLI	16	0.125	0.007
SXT	0.25/4.75	0.25/4.75	0.5
RIF	0.125	0.125	0.4
NIT	16	16	0.8

FOX, Cefoxitin; OXA, Oxacillin; GEN, Gentamicin; AMK, Amikacin; AZM, Azithromycin; ERY, Erythromycin; CLI, Clindamycin; SXT, Trimethoprim-sulfamethoxazole (Co-trimoxazole); RIF, Rifampicin; NIT, Nitrofurantoin.

Supplementary Dataset S1

Information of clinical and public isolates included in this study. Excel Sheet 1, Genotypic information and raw MIC data for 17 antimicrobials tested on 105 EIA-MRSA isolates. Excel Sheet 2, Accession numbers of 83 ST764 and 1,042 ST22 public *S. aureus* genomes included in this study.
